# *In vitro* antioxidant and antihyperlipidemic activities of *Bauhinia variegata* Linn

**DOI:** 10.4103/0253-7613.58513

**Published:** 2009-10

**Authors:** G.P. Rajani, Purnima Ashok

**Affiliations:** Department of Pharmacology, K. L. E. Society's College of Pharmacy, Bangalore, India

**Keywords:** Antihyperlipidemic, antioxidant, *Bauhinia variegata* linn, triton

## Abstract

**Objectives:**

To evaluate the ethanolic and aqueous extracts of *Bauhinia variegata* Linn. for *in vitro* antioxidant and antihyperlipidemic activity.

**Materials and Methods:**

Ethanolic and aqueous extracts of the stem bark and root of *B. variegata* Linn. were prepared and assessed for *in vitro* antioxidant activity by various methods namely total reducing power, scavenging of various free radicals such as 1,2-diphenyl-2-picrylhydrazyl (DPPH), super oxide, nitric oxide, and hydrogen peroxide. The percentage scavenging of various free radicals were compared with standard antioxidants such as ascorbic acid and butylated hydroxyl anisole (BHA). The extracts were also evaluated for antihyperlipidemic activity in Triton WR-1339 (iso-octyl polyoxyethylene phenol)-induced hyperlipidemic albino rats by estimating serum triglyceride, very low density lipids (VLDL), cholesterol, low-density lipids (LDL), and high-density lipid (HDL) levels.

**Result:**

Significant antioxidant activity was observed in all the methods, (*P* < 0.01) for reducing power and (*P* < 0.001) for scavenging DPPH, super oxide, nitric oxide, and hydrogen peroxide radicals. The extracts showed significant reduction (*P* < 0.01) in cholesterol at 6 and 24 h and (*P* < 0.05) at 48 h. There was significant reduction (*P* < 0.01) in triglyceride level at 6, 24, and 48 h. The VLDL level was also significantly (*P* < 0.05) reduced from 24 h and maximum reduction (*P* < 0.01) was seen at 48 h. There was significant increase (*P* < 0.01) in HDL at 6, 24, and 48 h.

**Conclusion:**

From the results, it is evident that alcoholic and aqueous extracts of *B. variegata* Linn. can effectively decrease plasma cholesterol, triglyceride, LDL, and VLDL and increase plasma HDL levels. In addition, the alcoholic and aqueous extracts have shown significant antioxidant activity. By the virtue of its antioxidant activity, *B. variegata* Linn. may show antihyperlipidemic activity.

## Introduction

Oxidation is one of the destructive processes, wherein it breaks down and damages various molecules. Oxygen via its transformation produces reactive oxygen species (ROS) such as super oxide, hydroxyl radicals, and hydrogen peroxide. They provoke uncontrolled reactions.[1] Molecular oxygen is an essential component for all living organisms, but all aerobic species suffer from injury if exposed to concentration more than 21%.[2]

Free radicals attack and induce oxidative damage to various biomolecules including proteins, lipids, lipoproteins, and DNA.[2,3] The body possesses several defense systems comprising enzymes and radical scavengers.[1] Some of them constitute the repair systems for biomolecules that are damaged by the attack of free radicals.[3]

Antioxidants are compounds that act as inhibitors of the oxidation process and are found to inhibit oxidant chain reactions at small concentrations and thereby eliminate the threat of pathological processes.[1] Phenolic compounds present in medicinal plants have been reported to posses powerful antioxidant activity.[2] Flavanoids are a major class of phenolic compounds present in medicinal plants and are found to have a potential role in prevention of various diseases through their antioxidant activity.[4]

*Bauhinia variegata* Linn. (Ceasalpiniaceae) is a medium-sized deciduous tree found throughout India. It is traditionally used in bronchitis, leprosy, and tumors. The stem bark is used as astringent, tonic, and anthelmintic.[5,6] Infusion of the leaves is used as a laxative and for piles. Dried buds are used in the treatment of worm infestations, tumors, diarrhea, and piles.[7] The stem bark is used in ayurveda for its antidiabetic activity.[8] So far, the stem bark has been investigated and reported to have antitumor,[9,10] antibacterial, antifungal, antiulcer, and hepatoprotective activity.[11] Flavanone glycoside from root is reported to have anti-inflammatory activity.[12] The stem bark is reported to contain 5,7 dihydroxy and 5,7 dimethoxy flavanone-4-O-α-L rhamnopyrosyl-β-D-glycopyranosides, Kaempferol-3-glucoside, lupeol, and betasitosterol. Seeds contain protein, fatty oil-containing oleic acid, linoleic acid, palmitic acid, and stearic acid. Flowers contain cyanidin, malvidin, peonidin, and kaempferol. Root contains flavanol glycosides.[13–17]

Since polyphenolic compounds are present in the ethanolic and aqueous extracts of stem bark and root of *B. variegata* Linn., it was thought that it would be worthwhile to evaluate the plant for antioxidant activity. Lipids are one of the most susceptible targets of free radicals.[3] This oxidative destruction is known as lipid peroxidation and may induce many pathological events. Apart from antioxidant studies, the present study therefore also involves evaluation of antihyperlipidemic activity.

## Materials and Methods

### Plant material and extraction

The stem bark and root of *B. variegata* Linn. were procured and authenticated from Regional Research Institute, Bangalore. The authenticated stem bark and root were dried in shade and powdered coarsely. Extraction was done according to standard procedures using analytical grade solvents. Coarse powders of the root (1 kg) and stem bark (1.1 kg) were separately Soxhlet extracted with 90% ethanol. The aqueous extract was prepared using the same marc by the process of maceration. The extracts obtained were concentrated under reduced pressure to yield the ethanolic extract of stem bark and root (4.3 and 4.2%, respectively) and the aqueous extract of stem bark and root (2.4% each).

### Preparation of test solution

The various extracts such as *B. variegata* stem alcoholic (BVSA), stem water (BVSW), root alcohol (BVRA), and root water (BVRW) extracts at various concentrations were prepared in water and used for *in vitro* antioxidant studies. Pilot studies were carried out for the ethanolic and aqueous extracts of stem bark and root of *B. variegata* for *in vitro* antioxidant studies and the concentrations at which the extracts gave good antioxidant activity was selected.

### Animals

Albino male Wistar rats weighing between 150 and 200 g were procured from registered breeders. The animals were housed under standard conditions of temperature (25 ±2°C) and relative humidity (30-70%) with a 12:12 light-dark cycle. The animals were fed with standard pellet diet and water *ad libitum*. Approval of the Institutional Animal Ethics Committee (IAEC) of K.L.E. Society's College of Pharmacy, Bangalore, was obtained.

### Acute toxicity studies

Acute toxicity studies for aqueous and ethanolic extracts of *B. variegata* Linn. were conducted as per OECD guidelines 423[18] using albino Wistar rats. Each animal was administered the aqueous solution of the extract by oral route. The animals were observed for any changes continuously for the first 2 h and upto 24 h for mortality.

### Antioxidant studies

The ability of the extracts to scavenge hydrogen peroxide,[19] DPPH (1,2-diphenyl-2-picrylhydrazyl) radical,[19] nitric oxide,[20,21] superoxide radical,[4] and its reducing power[19] was determined at different concentrations.

Butylated hydroxy anisole (BHA) and ascorbic acid were used as standards for the various *in vitro* antioxidant studies. The percentage scavenging of various radicals were calculated using the following formula:

% Radical scavenged=A0-A1A0

where A_0_ is absorbance of the free radical alone and A_1_ is absorbance of free radical in the presence of extract/standard. All the experiments were performed in triplicate.

### Antihyperlipdemic activity

The method of Tamasi *et al*.[22] was used for evaluation of antihyperlipidemic activity. Albino Wistar rats weighing between 190 and 250 g were assigned to various groups of six animals each. Animals were fasted for 16 h prior to the experiment with water *ad libitum*. The various extracts, *B. variegata* stem-water extract (BVSW), ethanolic extract (BVSA), *B. variegata* root-water extract (BVRW), and ethanolic extract (BVRA) each at doses of 200 and 400 mg/kg body weight, simvastatin at 4 mg/kg and fenofibrate at 20 mg/kg, were administered p.o. to groups II to XI, respectively. Group I served as control. On the day of the experiment, the animals of the groups II-XI received the respective drugs by oral route. Simultaneously, all the animals received Triton WR-1339 at 100 mg/kg body weight by intraperitoneal route. The control animals were given only Triton WR-1339 at 100 mg/kg body weight. Serum cholesterol, triglyceride, and HDL were estimated at 6, 24, and 48 h using AGAPPE diagnostic kits. Blood samples were withdrawn by retroorbital puncture. Total cholesterol was estimated by CHOD-PAP methodology, Triglycerides by GPO-PAP methodology, and HDL by the precipitation method using phosphotungstate magnesium acetate reagent.

VLDL was calculated using the formula,VLDL=Triglycerides5

LDL cholesterol was calculated as

LDL=Total Cholesterol -HDL−Triglycrides)5.

### Chemicals

The chemicals DPPH (1,2-diphenyl-2-picrylhydrazyl), N-(1-Naphthyl) ethylenediamine dihydrochloride, Triton WR-1339, NADH, SNP, phenazine methosulphate, trichloro acetic acid, and potassium ferricyanide were purchased from Sigma Chemicals, St Louis, MO, USA. All other chemicals and reagents used were of analytical grade. UV-1700 Shimadzu UV-Vis spectrophotometer was used for *in vitro* anti-oxidant studies.

### Statistical analysis

All the values are presented as mean α SD. Data were statistically analyzed by one-way ANOVA followed by *post hoc* test; *P* values < 0.05 were considered as statistically significant. Linear regression analysis was used for calculation of IC_50_.

## Results

### Acute toxicity studies

There was no mortality and noticeable behavioral changes in all the groups tested. The aqueous and ethanolic extracts of stem and root of *B. variegata* Linn. were found to be safe upto 2000 mg/kg body weight.

### Hydrogen peroxide scavenging activity

**Figure 1:**
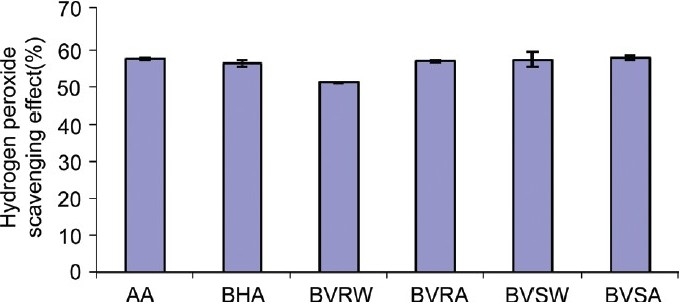
Comparison of hydrogen peroxide scavenging activity of 10 μg/ml of AA, BHA, BVRW, BVRA, BVSW and BVSA; (n = 3) AA: Ascorbic acid, BHA: Butylated hydroxy anisole; BVRW, BVRA, BVSW, BVSA: *Bauhinia variegata* root water, ethanolic and *Bauhinia variegata* stem water and ethanolic extracts, respectively

At 10 μg/ml concentration, BVSW, BVSA, and BVRA produced H_2_O_2_ scavenging activity comparable (*P* < 0.05) to that of the standards BHA and ascorbic acid, BHA, BVRW, BVRA, BVSW, and BVSA were found to have IC_50_ (mean ± SD) of 9.917 ± 0.01, 10.95 ± 0.03, 11.74 ± 0.21, 10.78 ± 0.17, 10.23 ± 0.11, 9.85 ± 0.03, respectively [Figure 1 and Table 1].

**Table 1:** Free radical scavenging activity of different extracts of *Bauhinia variegata* Linn

*Test/standard group*	IC_50_*values ± SD (μg/ml) for free radical scavenging activity*			
	
	*Hydrogen peroxide*	*DPPH*	*Nitric oxide*	*Super oxide*
Ascorbic acid	9.91 ± 1.03	30.55 ± 1.12	—	—
BHA	10.95 ± 2.11	29.11 ± 1.03	368.00 ± 2.60	435.40 ± 7.78
BVRW	11.74 ± 2.98	37.73 ± 1.37	478.80 ± 3.40	502.10 ± 8.00
BVRA	10.78 ± 1.70	36.01 ± 1.25	415.20 ± 2.88	445.30 ± 4.05
BVSW	10.23 ± 1.11	45.85 ± 2.49	405.80 ± 5.43	481.30 ± 5.00
BVSA	9.85 ± 0.93	30.50 ± 1.61	362.90 ± 3.80	414.60 ± 6.22

BVRW, BVRA, BVSW, BVSA: *Bauhinia variegata* root water, ethanolic and *Bauhinia variegata* stem water and ethanolic extracts, respectively; AA: Ascorbic acid, BHA: Butylated hydroxy anisole

### DPPH radical scavenging activity

The various extracts produced significant DPPH radical scavenging activity from 10 μg/ml. The IC_50_ (mean ± SD) of ascorbic acid, BHA, BVRW, BVRA, BVSW, and BVSA were found to be 30.55 ± 0.52, 29.11 ± 0.03, 37.73 ± 0.37, 36.10 ± 0.50, 45.85 ± 0.49, 30.50 ± 0.16, respectively [Table 1].

### Nitric oxide radical scavenging activity

Scavenging of nitric oxide by various extracts was found to be concentration dependent. Maximum inhibition of nitric oxide formation was produced by BVSA at concentration of 500 μg/ ml and had IC_50_ of 362.90 ± 3.80 as against 368.00 ± 2.60 for BHA [Tables 1 and 2].

### Total reducing power

From 50 μg/ml, all the extracts and standard-ascorbic acid and BHA showed reductive capabilities. At 500 μg/ ml, the reducing power of standards and extracts showed the following order: BHA > BVRA > BVSA > Ascorbic acid > BVSW > BVRW. Reducing power of BVSA and BVRA at 500 μg/ml was comparable (*P* < 0.05) to that of ascorbic acid [Table 2].

**Table 2:** Total reducing power, nitric oxide, and superoxide radical scavenging activity of different concentrations of AA, BHA, BVRW, BVRA, BVSW, and BVSA

*Group (mcg/ml)*	*Reducing power (Absorbance)*	*Nitric oxide scavenged (%)*	*Superoxide radical scavenged (%)*
AA			
100	0.09 ± 0.01	-	-
200	0.19 ± 0.02	-	-
300	0.61 ± 0.03	-	-
400	0.77 ± 0.01	-	-
500	1.47 ± 0.05	-	-
BHA			
100	0.13 ± 0.01^2a^	13.95 ± 0.41	20.51 ± 0.66
200	0.24 ± 0.01^2a^	20.86 ± 0.43	29.20 ± 0.45
300	0.80 ± 0.05^2a^	34.20 ± 0.18	38.36 ± 0.39
400	1.08 ± 0.02^2a^	40.11 ± 0.15	56.11 ± 0.12
500	1.75 ± 0.02^2a^	60.86 ± 0.41	65.78 ± 0.51
BVRW			
100	0.01 ± 0.00	11.84 ± 0.21	20.40 ± 0.10^1b^
200	0.05 ± 0.00	18.78 ± 0.13	29.50 ± 0.16^1b^
300	0.10 ± 0.01	28.68 ± 0.26	35.98 ± 0.17
400	0.24 ± 0.03	35.01 ± 0.16	39.46 ± 0.19
500	0.74 ± 0.03	52.61 ± 0.41	50.67 ± 0.45
BVRA			
100	0.04 ± 0.01	12.44 ± 0.61	33.44 ± 0.62^3b^
200	0.09 ± 0.01	21.65 ± 0.14^1b^	36.48 ± 0.50^3b^
300	0.40 ± 0.02	32.18 ± 0.13	39.00 ± 0.24^2b^
400	0.80 ± 0.01^1a^	39.86 ± 0.11^1b^	45.84 ± 0.20^1b^
500	1.65 ± 0.03^1a^	59.12 ± 0.12^1b^	55.90 ± 0.25
BVSW			
100	0.01 ± 0.00	14.86 ± 0.44^1b^	32.64 ± 0.67^3b^
200	0.04 ± 0.01	23.84 ± 0.54^1b^	34.21 ± 0.72^3b^
300	0.13 ± 0.02	30.14 ± 0.11	41.97 ± 0.12^2b^
400	0.21 ± 0.01	35.64 ± 0.61	45.95 ± 0.67^1b^
500	1.05 ± 0.03	54.80 ± 0.31	56.80 ± 0.94
BVSA			
100	0.05 ± 0.00	10.86 ± 0.84	30.40 ± 0.66^3b^
200	0.09 ± 0.00	20.68 ± 0.54^1b^	35.62 ± 0.86^3b^
300	0.47 ± 0.02	31.68 ± 0.31	50.10 ± 0.16^3b^
400	0.82 ± 0.01	40.58 ± 0.11^1b^	54.03 ± 0.24^1b^
500	1.60 ± 0.08^1a^	61.05 ± 0.38^1b^	56.90 ± 0.09

Values are mean ± SD, (n = 3); ^1^*P* < 0.05, ^2^*P* < 0.01, ^3^*P* < 0.001 as compared to ^a^AA, ^b^BHA. AA: Ascorbic acid; BHA: Butylated hydroxy anisole; BVRW, BVRA, BVSW, BVSA: *Bauhinia variegata* root water, ethanolic and *Bauhinia variegata* stem water and ethanolic extracts, respectively

### Superoxide anion radical scavenging activity

Superoxide anion radical generation was inhibited by BHA (standard) and extracts from 100 μg/ml. The various extracts produced significant superoxide radical scavenging activity in a concentration-dependent manner. BVSA showed the lowest IC_50_ value (414 ± 6.22) followed by BHA (435.40 ± 7.78).

### Antihyperlipidemic activity

Administration of Triton resulted in increase in serum levels of cholesterol, triglycerides, VLDL, and LDL. A significant reversal in serum levels of cholesterol, triglycerides, VLDL, and LDL levels was noticed in the animals treated with *B. variegata* Linn. root and stem extracts when compared with the control group [Tables 2 and 3].

**Table 3:** Effects of aqueous and ethanolic extracts of stem bark and root of *Bauhinia variegata* on total cholesterol and low density lipids levels in triton-induced hyperlipidemic rats

*Group*	6 *h*		24 *h*		48 *h*	
			
	*Serum cholesterol(mg/dl)*	*Serum LDL(mg/dl)*	*Serum cholesterol(mg/dl)*	*Serum LDL(mg/dl)*	*Serum cholesterol(mg/dl)*	*Serum LDL(mg/dl)*
Control	108.70 ± 1.86	88.27 ± 0.73	81.54 ± 2.04	59.06 ± 4.17	60.44 ± 2.71	45.53 ± 0.71
BVSW200	88.32 ± 1.35^2c^	58.88 ± 0.79^2c^	84.51 ± 1.38	65.04 ± 0.40^2c^	59.39 ± 3.24	29.75 ± 0.76^2a,2b,2c^
BVSW400	76.26 ± 0.97^2c^	43.71 ± 0.79^2c^	57.57 ± 1.17^2c^	23.56 ± 0.76^1a,2b,2c^	55.25 ± 3.09^1c^	23.65 ± 0.72^2a,2b,2c^
BVRW200	93.54 ± 0.90^2c^	73.35 ± 0.86^2c^	81.62 ± 1.17	57.54 ± 0.74^2c^	68.34 ± 3.24	49.23 ± 0.69
BVRW400	65.58 ± 0.55^2c^	33.55 ± 0.85^2c^	60.33 ± 0.92^2b,2c^	25.42 ± 1.42^1a,2b,2c^	57.98 ± 1.41^1a,1b,1c^	29.32 ± 0.70^2a,2b,2c^
BVSA200	66.53 ± 0.60^2c^	38.74 ± 0.95^2c^	67.44 ± 0.70^2c^	36.74 ± 0.80^2c^	55.38 ± 1.30^1a,1b,1c^	31.33 ± 0.72^2a,2b,2c^
BVSA400	63.48 ± 0.70^2c^	39.13 ± 0.89^2c^	59.52 ± 0.67^1b,1c^	26.60 ± 0.84^2b,2c^	53.44 ± 2.34^3a,3b,1c^	28.22 ± 0.67^2a,2b,2c^
BVRA200	61.35 ± 0.80^1b,2c^	39.49 ± 0.70^2b^	59.69 ± 0.58^1b,2c^	33.47 ± 1.31^1b,2c^	54.34 ± 1.38^3a,3b,1c^	30.64 ± 0.72^2a,2b,2c^
BVRA400	59.61 ± 0.74^1b,3c^	32.19 ± 0.89^2b^	60.27 ± 1.43^1b,2c^	26.41 ± 0.97^2b,2c^	51.52 ± 1.41^3a,3b,1c^	27.61 ± 0.80^2a,2b,2c^
Simvastatin	52.38 ± 0.93^3b,3c^	22.54 ± 1.01^3c^	51.20 ± 1.34^3b,3c^	22.75 ± 0.96^3b,3c^	62.29 ± 1.47	47.21 ± 0.68
Fenofibrate	61.16 ± 0.67^2c^	29.73 ± 0.90^2c^	62.28 ± 1.43^2c^	33.32 ± 0.94^2c^	62.38 ± 1.37	44.73 ± 0.47

Values are expressed as mean ± S.D. (n = 6). Cholesterol and LDL concentrations are estimated by the standard method and the values are expressed as mg/dl serum. ^1^*P* < 0.05, ^2^*P* < 0.01, ^3^*P* < 0.001, when compared with the ^c^control group; ^a^Simvastatin; ^b^Fenofibrate. BVRW, BVRA, BVSW, BVSA: *Bauhinia variegata* root water, ethanolic and *Bauhinia variegata* stem water and ethanolic extracts, respectively, at 200 and 400 mg/kg body weight

Simvastatin (standard) produced maximum cholesterol- and LDL-lowering effect at both 6 h and 24 h. BVRW 400, BVRA 200 and 400 and BVSA 200 and 400 produced a significant decrease in serum cholesterol and LDL levels, which was found to be significantly greater than the effects of f enofibrate (at 6 h, 24 h and 48 h) and simvastatin (at 48 h) [Table 2].

Maximum reduction of triglyceride and VLDL levels was produced by fenofibrate at 6 h. At 24 h and 48 h, aqueous and ethanolic extracts of *B. variegata* root and stem produced significant triglyceride- and VLDL-lowering effect which was comparable to that of fenofibrate and significantly greater than that of Simvastatin [Table 4].

**Table 4:** Effects of aqueous and ethanolic extracts of stem bark and root of *Bauhinia variegata* on total triglyceride and very low density lipids levels in triton-induced hyperlipidemic rats

*Group*	6 *h*		24 *h*		48 *h*	
			
	*Serum triglyceride(mg/dl)*	*Serum VLDL(mg/dl)*	*Serum Triglyceride(mg/dl)*	*Serum VLDL(mg/dl)*	*Serum Triglyceride(mg/dl)*	*Serum VLDL(mg/dl)*
Control	69.4 ± 0.71	13.72 ± 0.96	61.17 ± 0.84	12.10 ± 0.89	79.40 ± 1.34	15.81 ± 1.01
BVSW200	63.72 ± 1.36^2c^	12.66 ± 0.88^1a^	60.22 ± 0.82^1a^	20.17 ± 1.01	54.47 ± 0.66^2a,2b,2c^	10.84 ± 0.80^2a,2b,2c^
BVSW400	60.77 ± 1.39^2c^	12.10 ± 0.94^1a,1b,1c^	57.65 ± 0.70^2a,2c^	11.50 ± 0.88^1a,1b^	54.56 ± 0.74^2a,2b,2c^	10.89 ± 0.85^2a,2b,2c^
BVRW200	68.32 ± 0.52	13.66 ± 0.73	54.17 ± 0.66^2a,1b,2c^	10.71 ± 1.00^1a,1b^	59.68 ± 1.73^2a,1b,2c^	11.82 ± 0.86^2a,2b,2c^
BVRW400	63.43 ± 0.79^2c^	12.60 ± 0.87	54.55 ± 0.82^2a,1b,2c^	10.81 ± 0.82^1a,1b^	50.29 ± 0.69^2a,2b,2c^	10.18 ± 0.81^2a,2b,2c^
BVSA200	62.50 ± 0.87^2c^	12.46 ± 0.86	51.61 ± 0.6^2a,1b,2c^	10.43 ± 0.76^1a,1b,1c^	49.45 ± 0.66^2a,2b,2c^	09.75 ± 0.88^3a,3b,2c^
BVSA400	60.18 ± 1.01^2c^	11.90 ± 0.78^1c^	52.31 ± 0.67^2a,2c^	10.42 ± 0.71^1a,1b,1c^	43.67 ± 0.73^2a,3b,2c^	8.84 ± 0.83^3a,3b,2c^
BVRA200	67.48 ± 1.52^2c^	13.48 ± 0.85	58.46 ± 4.16^2a,1b,2c^	12.20 ± 0.89^1a,1b^	48.26 ± 0.71^3a,3b,2c^	12.13 ± 0.90^3a,3b,2c^
BVRA400	60.55 ± 0.70^2c^	12.23 ± 0.83^2c^	50.89 ± 1.06^2a,2b,2c^	10.56 ± 0.75^1a^	48.45 ± 0.68^3a,3b,2c^	09.60 ± 0.77^3a,3b,2c^
Simvastatin	63.73 ± 1.71^2c^	12.66 ± 0.86^1b^	63.24 ± 0.91	12.50 ± 0.86	65.34 ± 0.75^2b,2c^	13.19 ± 0.95^1b,2c^
Fenofibrate	55.20 ± 0.98^3a,2c^	11.34 ± 0.32^1a^	54.11 ± 0.71^3a,2c^	10.84 ± 0.64^1a^	58.58 ± 1.27^2a,2c^	11.91 ± 0.75^2a,2c^

Values are expressed as mean ± S.D. (n = 6). Triglyceride and VLDL concentrations are estimated by the standard method and the values are expressed as mg/dl serum; ^1^*P* < 0.05, ^2^*P* < 0.01, ^3^*P* < 0.001, when compared with the ^c^control group; ^a^Simvastatin; ^b^Fenofibrate; BVRW, BVRA, BVSW, BVSA: *Bauhinia variegata* root water, ethanolic and *Bauhinia variegata* stem water and ethanolic extracts, respectively, at 200 and 400 mg/kg body weight

Simvastatin, fenofibrate, and various extracts exscept BVRW and BVRA 200 mg/kg produced significant (*P* < 0.01) increase in serum HDL level at 6, 24, and 48 h when compared to control. At 6 h and 24 h all the extracts except BVRA 200 and BVRW 400 produced a significant (*P* < 0.01) increase in HDL level, which was significantly greater than that of simvastatin and fenofibrate. At 48 h BVSW 200 and 400, BVRA 200 and BVRW 200 produced significant (*P* < 0.01) increase in HDL level, which was significantly greater than that of simvastatin and fenofibrate [Table 5].

**Table 5:** Effects of aqueous and ethanolic extracts of stem bark and root of *Bauhinia variegata* on high-density lipid levels in triton-induced hyperlipidemic rats

*Group*	*Serum HDL (mg/dl)*		
	
	6 *h*	24 *h*	48 *h*
Control	34.20 ± 1.39	32.72 ± 1.00	31.16 ± 0.88
BVSW200	42.12 ± 0.87^1a,1b,2c^	40.73 ± 0.94^1a,1b,2c^	40.76 ± 0.89^1a,1b,2c^
BVSW400	44.53 ± 1.58^2a,2b,2c^	45.54 ± 0.94^2a,2b,2c^	42.61 ± 0.88^2a,2b,2c^
BVRW200	34.41 ± 1.84	34.48 ± 0.79^1c^	31.29 ± 1.01
BVRW400	44.79 ± 0.73^1a,1b,1c^	45.41 ± 0.91^2a,2b,2c^	39.01 ± 0.61^1a,1b,2c^
BVSA200	40.20 ± 0.75^2c^	42.25 ± 0.83^1a,2b,2c^	34.35 ± 0.69^2c^
BVSA400	40.40 ± 1.20^2c^	44.59 ± 0.97^2a,2b,2c^	34.41 ± 0.81^2c^
BVRA200	35.38 ± 1.50	37.54 ± 0.69^2c^	36.30 ± 1.46^1a,2c^
BVRA400	39.41 ± 0.57^2c^	44.08 ± 0.85^2a,2b,2c^	32.40 ± 1.42^2c^
Simvastatin	42.65 ± 0.76^1b,2c^	40.89 ± 0.87^1b,2c^	38.35 ± 1.00^1b,2c^
Fenofbrate	42.09 ± 0.74^1c^	39.73 ± 0.81^1a,2c^	39.45 ± 0.68^1a,2c^

Values are expressed as mean ± S.D. (n = 6). HDL concentrations are estimated by the standard method and the values are expressed as mg/dl serum; ^1^*P* < 0.05; ^2^*P* < 0.01; ^3^*P* < 0.001 when compared with the ^c^control group; ^a^Simvastatin; ^b^Fenofibrate; BVRW, BVRA, BVSW, BVSA: *Bauhinia variegata* root water, ethanolic and *Bauhinia variegata* stem water and ethanolic extracts, respectively, at 200 and 400 mg/kg body weight

## Discussion

The potentially reactive derivatives of oxygen ascribed as ROS such as superoxide radical, hydroxyl radical, and hydrogen peroxide are continuously generated inside the human body as a consequence of exposure to exogenous chemicals and/or a number of endogenous metabolic processes involving redox enzymes and bioenergetic electron transfer.[4] Owing to the ROS overproduction and/or inadequate antioxidant defense, there is upsurge of ROS and this culminates in oxidative stress. It is quite interesting to note that plants have good antioxidant ability and are safer than the synthetic antioxidants.[4] The antioxidant activity can be attributed to various mechanisms like prevention of chain initiation, binding of transition metal ion catalysts, decomposition of peroxides, reductive capacity, and radical scavenging activity.[4]

In the present study, five different antioxidant methods for evaluation of antioxidant activity have been used. Ethanolic and aqueous extracts of *B. variegata* root and stem produced significant antioxidant activity. This can be attributed to the flavonoids and other phytoconstituents present in the extracts. Stem ethanolic extract produced significantly greater antioxidant activity than root ethanolic and aqueous extracts.

Hyperlipidemia is one of the important risk factors involved in the development of cardiovascular diseases. Atherosclerosis and congestive heart diseases are strongly associated with disorders of lipid metabolism and plasma lipoproteins. Triton WR-1339-treated rats are considered to be a useful acute hyperlipidemic model associated with inactive lipoprotein lipase.[23] Triton WR-1339 acts as a surfactant to block the uptake of lipoprotein from the circulation by extra hepatic tissues resulting in an increase in the level of circulatory lipoproteins.[24] Triton WR-1339-induced hyperlipidemic rats treated with BVSW, BVSA, BVRW, and BVRA produced reversal of increase in serum cholesterol and triglycerides and LDL from the 6 h upto 48 h and VLDL from 24 h.

Ethanolic and aqueous extracts of *B. variegata* root and stem produced significant cholesterol and LDL lowering effect at 6, 24, and 48 h. This indicates that *B. variegata* not only reduces the synthesis of cholesterol, but may also reduces its metabolism. The extracts were found to be enriched in flavanoids and it is reported that flavanoids are found to inhibit HMG-CoA reductase activity.[23] It may be concluded that the cholesterol-lowering effect of *B. variegata* stem and root extracts may be due to inhibition of HMG-CoA reductase activity. Simvastatin being a specific HMG-CoA inhibitor produces its hypocholesterolemic activity by reducing cholesterol synthesis.

Increase in triglyceride level was evident in control animals due to inhibition of lipoprotein lipase (LPL) by Triton. Treatment with ethanolic and aqueous extracts of *B. variegata* resulted in reduction of triglyceride levels. It is likely that treatment with B. variegata might have lowered the serum triglyceride level by activating LPL. LPL is a prime enzyme related to triglyceride metabolism. Further VLDL levels were reduced significantly at 24 and 48 h.

The various extracts also showed protective action by increasing serum HDL level. The increased HDL facilitates the transport of triglyceride or cholesterol from serum to liver where it is catabolized and excreted out of the body. Significantly greater increase in HDL levels was produced by aqueous extracts than ethanolic extracts.

## Conclusion

The aqueous and ethanolic extracts of *B. variegata* Linn. have shown significant antioxidant activity. In the preliminary studies, it was found out that the aqueous and ethanolic extracts of *B. variegata* Linn. have shown promising antihyperlipidemic activity. *B. variegata* may partly owe its antihyperlipidemic activity to its antioxidant activity.
